# Correlation between general quality of life and oral health related quality in the mixed dentition

**DOI:** 10.1590/1807-3107bor-2024.vol38.0039

**Published:** 2024-05-13

**Authors:** Diego Patrik Alves CARNEIRO, Grazielle Araújo dos SANTOS, Caroline Nogueira de MORAES, Marcelo de Castro MENEGHIM, Silvia Amélia Scudeler VEDOVELLO

**Affiliations:** (a) Universidade Estadual de Campinas – Unicamp, Piracicaba Dental School, Department of Community Dentistry, Piracicaba, SP, Brazil.; (b) Fundação Herminio Ometto – FHO, Araras Dental School, Departament of Orthodontics, Araras, SP, Brazil.

**Keywords:** Quality of life, Oral health, Children, Dentition, Mixed

## Abstract

The aim of this study was to evaluate the convergence between the domains of the Autoquestionnaire Qualité de Vie Enfant image (AUQUEI) and the Child Perceptions Questionnaire (CPQ8-10) in the mixed dentition. A sample of 676 children aged 8 to 10 years responded to the health-related quality of life (HRQoL) and oral health-related quality of life (OHRQoL) questionnaires using the AUQUEI and the CPQ_8-10_, respectively. Clinical (dental caries and malocclusion) and socioeconomic variables were assessed. The validity of convergence between scores (total and per domain) of the two instruments was assessed by Spearman correlation analysis, considering that non-zero coefficient values represented a correlation between scores. The median was calculated to compare the scores of each questionnaire relative to the variables, and the nonparametric Mann-Whitney test was applied to determine statistically significant differences between the categories. A weak significant correlation (between 0.30 and 0.50) was observed between the domains and the total scores of instruments (p < 0.05), except for the leisure domain (p > 0.05). Participants with a lower family income had worse HRQoL (p < 0.05), and those with caries and malocclusion experience had worse OHRQoL (p < 0.05). In conclusion, the AUQUEI and CPQ_8-10_ instruments showed a weak correlation. Income and clinical variables had a negative impact on the AUQUEI and CPQ_8-10_, respectively.

## Introduction

Quality of life is used as a measure of satisfaction with life, influenced by social and economic factors and the state of general health.^1-3^ Socioeconomic factors, age, and education can have a decisive influence on the perception of satisfaction with life.^2,4^ Furthermore, oral health appears to have an impact on general health and, consequently, on quality of life^5^, as it can represent pain and/or suffering, change in eating habits, speech, and social interaction.^2-4,6^ Thus, to assess the general quality of life and the OHRQoL, irrespective of the instrument used, the social, environmental, political, and cultural context of each of them should be considered.^3^

Around the age of eight, children can identify physical characteristics related to their appearance based on criteria similar to those used by adults.^7-9^ Although some characteristics of occlusion during mixed dentition do not constantly configure the presence of malocclusion, it is during this phase that the most remarkable and most significant changes in children’s occlusion occur.^4,9^ Moreover, mixed dentition is the phase that allows most interceptive orthodontic procedures,^10^ which justifies understanding of the multidimensional aspects that are involved in this period.

The Child Perceptions Questionnaire (CPQ_8-10_)^11-13^ is widely used to assess children’s perception of the impacts of oral health problems on quality of life.^12^Whereas Autoquestionnaire Qualité de Vie Enfant Imagé (AUQUEI) evaluates the HRQoL based on the principle that this developing individual can express this condition subjectivity.^14^The questionnaire is based on the child’s satisfaction with the family, social activities, health, bodily functions, and separation from the family, identified in images that express different moods.^3,4,14,15^

The present study tested the hypothesis that the AUQUEI and CPQ_8-10_ domains are?/would be correlated. Therefore, the study aim was to correlate the AUQUEI and CPQ_8–10_ domains in mixed dentition modulated by socioeconomic and clinical variables.

## Methodology

The Research Ethics Committee approved the present study (#87570618.4.0000.5385). All participants and their parents/guardians were informed about the study objectives. The present study followed the STROBE statement for cross-sectional studies.^16^

A representative sample of children in the study age group was selected from public schools. Initially, 19 public schools were selected by random sampling, stratified according to the population of schoolchildren in the neighborhoods. Then, all volunteers in the age group of the schools selected were invited to participate. The sample was calculated using the EpiInfo software (Centers for Disease Control and Prevention, Atlanta, USA), considering a test power of 80%, a significance level of 5%, and a minimum odds ratio of 1.5. The final sample consisted of 676 children (345 girls and 331 boys).

The study included children in the mixed dentition stage determined by clinical examination.^4,17,18^ Individuals with systemic diseases, such as cerebral palsy or Down syndrome, complete primary, and permanent dentures, and previous or undergoing current orthodontic treatment were excluded since they did not meet the eligibility criteria. The final sample consisted of 676 children (345 girls and 331 boys).

### Data collect

The children were clinically evaluated inside the schools under natural light by a single calibrated evaluator. Before starting the data collection phase, complete training was carried out, with part of this period being used for the calibration process to verify the inter-examiner agreement. Based on the assessment of a gold standard rater, the inter-rater Kappa coefficient was greater than 0.91 and 0.93 for the clinical assessments of dental caries and malocclusion, respectively.

The presence of dental caries was diagnosed using the dmfd and DMFT-D indices according to the criteria recommended by the World Health Organization (WHO).^19^ The results of dental caries were dichotomized and classified into no experience of dental caries (dmfd/DMF-D = 0) and experience of dental caries (dmfd/DMF-D ≥ 1).^4, 20^

Malocclusion in the mixed dentition was evaluated based on the criteria of Grabowski et al.^21^ The position of the upper canine determined the anteroposterior relationship in the intercuspation relationship between the lower canine and the primary first molar, configuring a Class I canine. Deviations from normal positioning were defined as Class II, Class III, and asymmetry. To define overjet, the distance between the buccal surface of the mandibular incisor and the maxillary incisal edge was considered. Overjet was normal when the distance was between 0 and 2mm, increased by > 2 mm, and decreased by <0mm; the latter configured the presence of anterior crossbite. The anterior vertical relationship (overbite) was defined as normal when the maxillary incisors covered up to 2 mm of the mandibular incisors, overbite when the maxillary incisors covered more than 2 mm of the mandibular incisors and anterior open bite, when this distance between the incisors had values ≤ 0 mm. The posterior transverse relationship was classified as normal when the maxillary arch had transverse dimensions compatible with the mandibular arch. Therefore, the presence of posterior crossbite, unilateral or bilateral, or scissor bite configured the presence of posterior crossbite.^21^ Children diagnosed with at least one of the above criteria outside the normal range were classified as having malocclusion.^4,7^

AUQUEI determined the HRQoL assessment.^15^ The AUQUEI is composed of 26 questions about the child’s satisfaction with family, social activities, health, bodily functions, and separation, divided into four domains: autonomy (6 questions), leisure (6 questions ), roles (6 questions) and family (8 questions). The scale uses images of four faces that express different emotional states, with possible responses: very unhappy (score 0), unhappy (score 1), happy (score 2), and very happy (score 3). The domains were scored individually, and by the sum of the total scores that could vary from 0 to 78, and the lower the value, the worse the HRQoL.^3,4,15^

The CPQ_8-10_^11,12^ was used to evaluate the OHRQoL. The CPQ_8-10_ has 25 questions, divided into four domains: oral symptoms (5 questions), functional limitations (5 questions), emotional well-being (5 questions), and social well-being (10 questions). Response scores based on the frequency of events are established by a 5-point Likert scale: never (score 0); once or twice (score 1); sometimes (score 2); frequently (score 3) and every day or almost every day (score 4). The domains were scored individually, and by the total score, which could range from 0 to 100. Higher scores indicated a greater impact on OHRQoL.^18^

Socioeconomic data is considered information derived from the family environment. Parents and/or guardians answered a questionnaire containing questions about income and education and information about the number of people who lived in the same family environment.

### Data analysis

The sample was divided into four groups to compare the instruments, considering the better and worse quality of life. Values lower than the AUQUEI median indicated worse HRQoL, and values higher than the CPQ_8-10_ median indicated worse HRQoL: G1: lower AUQUEI scores and lower CPQ_8-10_ scores; G2: lower AUQUEI scores and higher CPQ_8-10_ scores; G3: higher AUQUEI scores and lower CPQ_8-10_ scores and G4: higher AUQUEI scores and higher CPQ_8-10_ scores. The absolute and relative frequencies of cases were calculated for each group. The validity of convergence between the scores (total and by domain) of the two instruments was evaluated by Spearman’s correlation analysis, considering that coefficient values other than zero represent a correlation between the scores. The parameters for the correlation coefficient were 0.90–1.00 (very strong correlation), 0.70–0.90 (strong correlation), 0.50-0.70 (moderate correlation), 0.30–0.50 (weak correlation) and 0.00–0.30 (very weak correlation).^22^

For comparison between the scores of each questionnaire (AUQUEI and CPQ_8-10_) as regards sociodemographic and clinical variables, the median was calculated, and the non-parametric Mann-Whitney test was applied to determine statistically significant differences between categories. Analyses were performed using the R program (R Foundation for Statistical Computing, Vienna, Austria) with a significance level of 5%.

## Results

Table 1 shows the descriptive data of the median responses by domain and the total score of the two instruments. The results showed that 48.1% of children reported worse HRQoL, considering the AUQUEI score. When the AUQUEI domains were evaluated, 49.7% of the children reported impact on the Family item. Relative to the OHRQoL, 50.6% of the children reported impact, and the Functional limitations domain was the one most impacted (57.8%).

**Table 1:** Descriptive data of the median responses of the two instruments.

AUQUEI^1^					

Domains	Autonomy	Leisure	Functions	Family	Total score
Median	12	14	13	13	52
n (%)	240 (35.5)	280 (41.4)	289 (42.8)	336 (49.7)	325 (48.1)
CPQ_8-10_^2^					
Domains	Oral symptoms	Functional limitations	Emotional well-being	Social well-being	Total score
Median	8	3	4	3	19
n (%)	361 (53.4)	391 (57.8)	339 (50.1)	356 (52.7)	342 (50.6)

AUQUEI^1^: Values lower than the median indicate worse HRQoL; autonomy, leisure, and family domains could present scores between 0 and 18; domain function scores between 0 and 24; total scores between 0 and 78. CPQ_8-10_^2^: Values higher than the median indicate worse OHRQoL; domain oral symptoms, functional limitations, and emotional well-being could present scores between 0 and 20; domain social well-being scores between 0 and 40; total scores between 0 and 100.

Table 2 presents the absolute and relative frequencies of comparison between instruments about?/between the groups. In group G1, 24.4% of children had worse HRQoL and better HRQoL; in G4, 18.2% (G4) had better HRQoL and worse HRQoL. This showed that 42.6% (G1+G4) of the children presented divergent results in the instruments. In G2, 30.0% presented worse HRQoL and OHRQoL; in G3, 27.4% presented better HRQoL and OHRQoL, indicating that 57.4% (G2+G3) presented concordant results in the instruments.

**Table 2:** Absolute and relative frequencies of comparisons between instruments.

^2^AUQUEI	^3^CPQ_11-14_	

	Frequency (%)	

	Lower (≤^1^19) - better	Higher (>^1^19) - worse
Lower (≤^1^52) - worse	165 (24.4%) – G1	203 (30.0%) – G2
Higher (>^1^52) - better	185 (27.4%) – G3	123 (18.2%) – G4

^1^Sample median. ^2^Lower scores mean worse HRQoL, ranging from 0 to 78. ^3^Higher scores mean worse OHRQoL, ranging from 0 to 100.

Table 3 presents the results of correlation between the domains and the total scores of the AUQUEI and CPQ_8-10_ questionnaires. Based on the results, except for the “Leisure” domain, a weak significant correlation between the domains and total scores of the two instruments was observed in the other domains (p < 0.05).

**Table 3:** Spearman correlation* analysis between the AUQUEI and CPQ8-10 instrument.

AUQUEI	CPQ_8-10_				

	Oral symptoms	Functional Limitations	Emotional well-being	Social well-being	Total score

	r (p-value)	r (p-value)	r (p-value)	r (p-value)	r (p-value)
Autonomy	-0.09 (0.0195)	-0.21 (< 0.0001)	-0.17 (<0.0001)	-0.13 (0.0010)	-0.18 (< 0.0001)
Leisure	-0.01 (0.8636)	-0.08 (0.0404)	-0.06 (0.0919)	-0.05 (0.1936)	-0.06 (0.1096)
Functions	-0.15 (< 0.0001)	-0.16 (< 0.0001)	-0.15 (< 0.0001)	-0.12 (0.0024)	-0.17 (< 0.0001)
Family	-0.09 (0.0178)	-0.13 (0.0007)	-0.12 (0.0025)	-0.09 (0.0194)	-0.13 (0.0009)
Total score	-0.14 (0.0002)	-0.22 (<0.0001)	-0.20 (< 0.0001)	-0.16 (< 0.0001)	-0.22 (< 0.0001)

*Correlation coefficient: 0.90-1.00 (very strong correlation), 0.70–0.90 (strong correlation), 0.50–0.70 (moderate correlation), 0.30–0.50 (weak correlation) and 0.00–0.30 (very weak correlation).

Table 4 shows the comparison between the AUQUEI and CPQ8-10 scores relative to the sample socioeconomic, demographic, and clinical characteristics. The results showed a significant difference in AUQUEI as a function of family income (p < 0.05). Children with lower income families had worse HRQoL (p < 0.05). CPQ8-10 showed a significant difference in the OHRQoL for the clinical variables (experience of caries and malocclusion). Children with dental caries experience and malocclusion reported worse OHRQoL (p < 0.05).

**Table 4:** Analysis of AUQUEI and CPQ8-10 scores according to sociodemographic and clinical variables.

Variable	n	^1^AUQUEI		^2^CPQ_8-10_	

		median	p-value	median	p-value
Sex					
Male	331	52.0	0.9277	19.0	0.6316
Female	345	52.0		18.0	
Income (salary)					
Up to 1	342	51.0	0.0016	19.0	0.6567
Above 1	334	53.0		18.0	
Residents					
Up to 3	155	52.0	0.9141	21.0	0.0585
Above 3	521	52.0		18.0	
Parents’ education					
Up to 8 years	452	52.0	0.6627	18.0	0.7811
More than 8 years	224	52.0		19.0	
Caries experience					
Without	238	52.0	0.4315	16.5	0.0045
With	438	52.0		20.0	

## Discussion

Placing value on oral health as a parameter to improve quality of life has been highlighted in studies of all ages. In this study, instruments to assess HRQoL and OHRQoL (AUQUEI and CPQ_8-10_) were correlated based on data collected in a mixed dentition occlusal stage sample. The mixed dentition is a stage with many biological events and occlusal changes that reflect children’s oral health, especially if we consider self-esteem and bullying episodes^7,10^. These factors justify the purpose of understanding the relationship between oral and general health quality. The present study is the first to evaluate the correlation between HRQoL and OHRQoL instruments in a mixed dentition population.

Studies related to mixed dentition have shown that age, cultural environment, and the social context can modify the OHRQoL of children.^18^Quality of life in this age group may also directly impact adherence to orthodontic treatment. However, assessing the impact of oral health on individuals’ quality of life is challenging, especially among children. In this sense, the professional may have difficulty identifying the main orthodontic complaint.

Our findings showed a weak correlation between AUQUEI and the CPQ_8-10_, reinforcing the hypothesis that the instruments have different constructs.^3^ Although the AUQUEI and CPQ_8-10_ measure the quality of life, they have structural differences in their design and domains, which may explain the lack of convergence between the instruments.^3,23-25^ Previous studies^3,23,24^have used a similar methodology to assess the positive or negative correlation between specific (OHRQoL) and generic (HRQoL) instruments with inverse worst and better score scales; however, in other age groups.

The social determinants of health are associated with quality of life^2-4^since individuals with lower family income had worse OHRQoL.^26,27^The clinical variables studied confirmed this statement. Children with experience of dental caries and malocclusion had worse OHRQoL scores.^26,28-31^ Therefore, it seemed clear that the perception of quality of life measured by specific or generic questionnaires could be associated with the social determinants discussed.

It is important to emphasize that our study did not evaluate the general health conditions, but only problems related to oral health and sociodemographic factors of its participants and families. Future studies should include general health conditions and the development of other instruments for assessing the HRQoL at an earlier age. Finally, our findings reinforced the importance of specific instruments for all age groups and the need for subjective assessments to implement and evaluate community health strategies.

## Conclusion

The AUQUEI and CPQ_8-10_ instruments showed a weak correlation. The income and clinical variables negatively impacted the AUQUEI and the CPQ, respectively.
